# Kinematic Changes in a Mouse Model of Penetrating Hippocampal Injury and Their Recovery After Intranasal Administration of Endometrial Mesenchymal Stem Cell-Derived Extracellular Vesicles

**DOI:** 10.3389/fncel.2020.579162

**Published:** 2020-09-10

**Authors:** Lilia Carolina León-Moreno, Rolando Castañeda-Arellano, Irene Guadalupe Aguilar-García, María Fernanda Desentis-Desentis, Elizabeth Torres-Anguiano, Coral Estefanía Gutiérrez-Almeida, Luis Jesús Najar-Acosta, Gerardo Mendizabal-Ruiz, César Rodolfo Ascencio-Piña, Judith Marcela Dueñas-Jiménez, Jorge David Rivas-Carrillo, Sergio Horacio Dueñas-Jiménez

**Affiliations:** ^1^Laboratory of Neurophysiology, Department of Neuroscience, University Center for Health Sciences, University of Guadalajara, Guadalajara, Mexico; ^2^Department of Biomedical Sciences, University Center of Tonala, University of Guadalajara, Guadalajara, Mexico; ^3^Laboratory of Tissue Engineering and Transplant, Department of Physiology, cGMP Cell Processing Facility, University Center for Health Sciences, University of Guadalajara, Guadalajara, Mexico; ^4^Department of Computer Sciences, University Center of Exact Sciences and Engineering, University of Guadalajara, Guadalajara, Mexico

**Keywords:** kinematic analysis, extracellular vesicles, endometrial mesenchymal stem cells, penetrating hippocampal injury, neuronal preservation

## Abstract

Locomotion speed changes appear following hippocampal injury. We used a hippocampal penetrating brain injury mouse model to analyze other kinematic changes. We found a significant decrease in locomotion speed in both open-field and tunnel walk tests. We described a new quantitative method that allows us to analyze and compare the displacement curves between mice steps. In the tunnel walk, we marked mice with indelible ink on the knee, ankle, and metatarsus of the left and right hindlimbs to evaluate both in every step. Animals with hippocampal damage exhibit slower locomotion speed in both hindlimbs. In contrast, in the cortical injured group, we observed significant speed decrease only in the right hindlimb. We found changes in the displacement patterns after hippocampal injury. Mesenchymal stem cell-derived extracellular vesicles had been used for the treatment of several diseases in animal models. Here, we evaluated the effects of intranasal administration of endometrial mesenchymal stem cell-derived extracellular vesicles on the outcome after the hippocampal injury. We report the presence of vascular endothelial growth factor, granulocyte–macrophage colony-stimulating factor, and interleukin 6 in these vesicles. We observed locomotion speed and displacement pattern preservation in mice after vesicle treatment. These mice had lower pyknotic cells percentage and a smaller damaged area in comparison with the nontreated group, probably due to angiogenesis, wound repair, and inflammation decrease. Our results build up on the evidence of the hippocampal role in walk control and suggest that the extracellular vesicles could confer neuroprotection to the damaged hippocampus.

## Introduction

Hippocampus is a susceptible brain structure to neurodegenerative diseases (Nikonenko et al., 2009), mainly related to memory, learning, and spatial orientation (Andersen et al., 2007). However, it is related to the sensory-motor system in murine models (Bland, 2009; López Ruiz et al., 2015; Bland et al., 2016), and its role in the gait control has been well known (Beauchet et al., 2019). Speed changes due to neuron loss in dorsal rat hippocampus and the interruption of the sensory-motor circuitry are described (López Ruiz et al., 2015). Lesions or degeneration in this structure impairs a variety of motor and behavioral tasks in mice (Cernak et al., 2014; Tucker et al., 2017; Hwang et al., 2018; Khan et al., 2018), including speed locomotion changes. Coordination between the hippocampus and lateral septum occurs through theta oscillations and facilitates theta inputs to the lateral hypothalamus, which in turn controls speed locomotion (Bender et al., 2015). Nucleus incertus stimulation augments theta power in the hippocampus and accelerates locomotion speed. This is mediated, in part, through the medial septum nucleus pathway to the hippocampus (Lu et al., 2020). However, less is known about locomotion kinematics disruption after hippocampal injury in mice, mainly because of the lack of a methodology useful to analyze the data. Our first aim was to study kinematic changes in a mouse model of penetrating hippocampal injury with our new developed methodology. To date, there is no model that affects hippocampus without damage of the cortex. This model reflects hippocampal injury caused by mild to moderate traumatic brain injury (TBI) in the hippocampus. TBI could affect connectivity of hippocampus (Girgis et al., 2016; Yan et al., 2016) probably due to cellular and synaptic loss secondary to the trauma (Atkins, 2011). Neurodegenerative pathologies and dementia could develop after TBI (Shively et al., 2012). Gait variability has been described for normal aging and patients with mild cognitive impairment, dementia, or Alzheimer disease (Montero-Odasso et al., 2009; Papp et al., 2014; O’Shea et al., 2016; Beauchet et al., 2019). To date, there is no effective treatment for TBI or any of the subsequent pathologies. Currently, mesenchymal stem cells (MSCs) are widely used in both research and cell-based therapy for various human diseases, including neurological and neurodegenerative disorders (Jeong et al., 2014; Gómez-Virgilio et al., 2018). Human endometrium has been reported as an attractive and accessible source for MSCs (Meng et al., 2007; Musina et al., 2008; Gargett et al., 2009). Endometrial MSCs (eMSCs) can be obtained without invasive procedures (Du et al., 2016) and possess a higher proliferation rate and migration capacity compared with MSCs obtained from other sources (Alcayaga-Miranda et al., 2015; Du et al., 2016). These cells can be differentiated into several lineages, including neurons (Meng et al., 2007). In this study, we use eMSCs to expand the knowledge of its effects on brain injuries. MSCs secrete both growth and anti-inflammatory factors through the secretion of extracellular vesicles (EVs; Drago et al., 2013; Kupcova Skalnikova, 2013). The EVs derived from MSCs (EV–MSCs) are round, and small vesicles originated from endosomes that are membrane-bound. EVs contain regenerative and neuroprotective molecules (Lötvall et al., 2014; Pachler et al., 2017), which could induce angiogenesis and neurovascular plasticity and improve neurobehavioral performance (Doeppner et al., 2015; Paquet et al., 2015; Chen et al., 2016; Kim et al., 2016; Zhang et al., 2017; Williams et al., 2019). Furthermore, intranasal (IN) administration route allows MSC–EVs to reach the brain directly. After 24 h of IN administration, EVs had been found in the cell cytoplasm of neurons, glia and endothelium in the olfactory bulb, cerebral cortex, dorsal and ventral striatum, thalamus, hypothalamus, brainstem, basal forebrain, and dorsal hippocampus (Long et al., 2017; Ezquer et al., 2019), grouped inside the cytoplasm of dentate hilar, CA3, and CA1 pyramidal and dentate granule neurons and nearby astrocyte and microglial processes (Long et al., 2017). In animal models, eMSC-derived EVs (eMSC–EVs) proved to be efficient for the treatment of hepatic failure (Chen et al., 2017), cardioprotection (Wang et al., 2017), diabetic wound healing (Dalirfardouei et al., 2019), Asherman syndrome (Saribas et al., 2019), and type 1 diabetes (Mahdipour et al., 2019). Further investigation is required to determine the role of eMSC–EVs in neuroprotection from direct damage in mouse brain tissue.

We show speed reduction and metatarsus kinematics changes after the hippocampal lesion. Further, behavioral outcomes improvements, restoration of locomotion speed and metatarsus kinematics, the decrease of pyknotic cells, and lesion area reduction were also observed after eMSC–EV treatment.

## Materials and Methods

All materials described below are available upon request to any interested researchers.

### Animals

Animal use and protocols were approved by the “Comité Insitucional de Cuidado y Uso de Animales de Laboratorio (CICUAL),” affiliated to the University of Guadalajara and endorsed to “Comisión Nacional de Bioética” (CONBIOÉTICA-14-CEI-002-20191003). All animals were treated following the Mexican Official Norm guidelines (NOM-062-ZOO-1999) and the National Institutes of Health Guide Publication No. 8023 (revised in 1996) for the Care and Use of Laboratory Animals. Adult male C57BL6 mice (20–25 g of weight) were used. We put the animals in groups of five, in plastic cages with a 12-h light–dark cycle at room temperature (22°C ± 2°C) and constant humidity. Water and food were available *ad libitum*. We used a minimal number of animals and minimized their suffering to obtain reliable results.

### Experimental Study Groups

We divided mice into three groups of five animals each: cortex-injured group (CI), hippocampus-injured without treatment group (HI), and hippocampus-injured with EV treatment group (HIEV). After surgery, we randomly divided hippocampus-injured mice into nontreated and treated groups. We used the CI group to determine the effect of sensorimotor cortex damage caused by penetrating injury. We performed kinematic and open-field (OF) locomotion analyses in the same animals. We made a histological analysis on these subjects, immediately after the last kinematic assay (7 days after the penetrating injury). We administered eMSC–EVs intranasally on Day 1 after injury in a dose of 500 μg of EV protein per kg of animal weight.

### Brain Injury Model

We used isoflurane gas at 5% for induction and 2–3% for anesthesia and performed surgeries under aseptic conditions. After verifying the absence of pain reflexes, we placed mice in a stereotaxic frame, and a head incision over the middle line was made. A cleft was drilled into the left parietal bone to expose the meninges, and a 0.5-mm-diameter sterile steel cannula was placed horizontally 1.2 and 2.5 mm behind Bregma, according to Paxinos and Franklin coordinates. We lowered a cannula to a 2-mm depth for the hippocampus-injured groups and to 1 mm in the cortex-injured group, displaced rostrally 1 mm (−1.5 from Bregma), and pulled out. We applied pressure over the injury to stop the bleeding. After the procedure, we sutured the scalp incision with 4–0 nylon sutures using lidocaine at 2%. After the surgery, we placed mice in a heat blanket for 30 min. Then, mice received antibiotics for 3 days (Enroxil 2 mg/kg) and analgesics (meloxicam 2 mg/kg).

### MSC Culture and Characterization

As xenograft tissue models had been successfully used, we isolated eMSCs from one adult human donor with written informed consent. These cells were provided by CryoVida, Banco de Células Madre Humanas, a stem cell bank certified and approved by COFEPRIS (license no. 18-TR-14-039-0002). Passage 2 cells were thawed at 37°C, washed with Hanks balanced salt solution (Sigma–Aldrich), and plated directly at a concentration of 7 × 10^6^ cells/75 cm^2^ in tissue culture flasks (Corning) with a xeno-free medium. All cells were cultured at 37°C in an incubator with 5% CO_2_. After cell confluence, the cells were continuously expanded under the same conditions, replacing the medium every 2–3 days. We used only cells in passages three to six and employed Accutase (Sigma–Aldrich) for cell passage. To evaluate the characteristics of the eMSC by flow cytometry, we determined the presence of surface MSC markers CD105, CD73, and CD90 and the absence of hematopoietic markers CD45 and CD34. The MSCs are sterile [negative for bacteria, yeast, fungi, mycoplasma (<10 colony-forming units), and endotoxin (<5 EU/kg/h)].

### Extracellular Vesicle Obtention and Characterization

For conditioned media obtention, we removed the xeno-free medium, and the cell layer washed with Hanks solution. We replaced the media for a protein-free solution and incubated the culture at 37°C and 5% of CO_2_. We recovered the medium at 48 h and was either stored at −80°C or used directly to isolate EVs. EVs were isolated from the cell culture media *via* multistep centrifugation at 4°C and ultrafiltration. We centrifuged cell culture media at 300 *g* for 3 min, and the supernatant was removed and centrifuged at 2,000 *g* for 10 min to remove small debris. The resulting media were further centrifuged at 10,000 *g* for 30 min. The supernatant is removed and placed in an Amicon Ultra-15 tube (30-kDa cutoff, Merck Millipore) and centrifuged at 5,000 *g* for 20 min. The obtained EVs were maintained at −80°C for further analysis.

We characterized the EVs from three independent experiments. We quantified EVs by measuring the total protein concentration using the micro-bicinchoninic acid protocol (Thermo Fisher Scientific) and analyzing particle size using dynamic light scattering (DLS) in a Zetasizer NANO ZS90 analyzer (Malvern), according to manufacturer’s instructions. The morphology of the EVs was assessed by transmission electron microscopy (TEM). We evaluated the characteristics of the isolated vesicles with the extraction of equal amounts of protein for Western blotting to detect the expression of Tsg101 (Santa Cruz Biotechnology).

We determine the presence of proinflammatory and anti-inflammatory cytokines of our interest: interleukin 17A (IL-17A), IL-17F, IL-23, IL-10, IL-6, IL-1β, tumor necrosis factor α (TNF-α), interferon γ (IFN-γ), and the following growth factors: granulocyte–macrophage colony-stimulating factor (GM-CSF) and vascular endothelial growth factor (VEGF) using the MILLIPLEX Multi-Analyte Profiling (MAP) Human Cytokine/Chemokine Kit (catalog no. HCYTOMAG-60K) and Human TH17 (catalog no. HTH17MAG-14K), run on a Luminex platform, according to manufacturer’s recommendations (Merck Millipore). For quality assurance, each sample was run twice, and the mean derived for each sample, using it as the index value. Kit-supplied quality controls and standards were run on each plate in duplicate and confirmed to fall within the expected range. Where an analyte level was below detection limits, we reported it as the lowest standard value. We analyzed the obtained fluorescence data with Beadview software.

### IN Administration of Extracellular Vesicles

We performed the procedure of IN administration as described elsewhere (Long et al., 2017; Ezquer et al., 2019), with minimal modifications. Briefly, anesthetized animals were placed in a supine position, and the ventral surface of the head and neck is maintained horizontal. We administered the eMSC–EV preparations *via* the olfactory pathway using a 10-μl Hamilton microsyringe, in aliquots of 5 μl, and alternating nostrils. We waited for 2 min between each administration. Each mouse received a total of 500 μg of EVs (released by 5 × 10^6^ eMSCs) protein per kilogram of animal weight diluted in 25 μl of EV preparation 24 h after the penetrating brain injury [1 day postinjury (DPI)] as described before for TBI and stroke animal models (Xin et al., 2013; Zhang et al., 2015, 2017).

### Neurological Outcome and Open-Field Tests

We used a modified Neurological Severity Score (mNSS) by a blinded investigator to determine the balance ability, motor functions, and sensory reflexes in mice as described before (Singh et al., 2018), with slight modifications: the prehensile test was incorporated to the 15-point scale (0–3 points), resulting in an 18-point scale in this study. We assigned a score of 0 to animals with normal behavior, and 18 is the score of the most severe neurological outcome. We tested mice before the injury (Day 0) and in days 1, 3, 5, and 7 postinjury.

We evaluated locomotor activity as well as anxiety, in an OF test before and 7 DPI. We used mice recorded before the injury (Day 0) as control for further comparisons. The OF arena was made of a wooden square box (32 × 45 cm) with a 30-cm-high wall. The floor is divided into 16 quadrants (11.25 × 8 cm) and subdivided into two imaginary squares called center and periphery. We consider the 12 quadrants adjacent to the walls as periphery. We individually placed mice in the center of the open field and allowed to explore the box for 10 min, cleaning the box using 70% ethanol and water after each mouse evaluation. We recorded mice using a Nikon camera with a resolution of 30 fps and analyzed them with the ImageJ software. We also determined the seconds the animal spent in the periphery of the open field. We analyzed the total distance traveled (cm) by mice and calculate the locomotion speed (cm/s). In all cases, we eliminated the time when mice remained still.

### Tunnel Walk Recordings

We obtained kinematic records before (Day 0) and after 7 DPI. We took mice recorded before the injury as a control group for further comparisons. We took video recordings of the animals while walking on a transparent Plexiglas tunnel (tunnel walk), using two synchronized cameras that record left and right hindlimbs simultaneously. We set the cameras to record at 240 fps with a resolution of 1,280 × 720 pixels. Postprocessing was applied to the resulting videos to remove distortion due to the lenses by estimation of homography matrix using four points on the image (Ascencio-Piña et al., 2020). We separate the steps of interest using the manual definition of instants corresponding to the beginning and end of the step. Manual annotation of the points of interest: knee, ankle, and metatarsus, was performed on each frame of the video for each step using a custom-made software, called “ratwalk” to obtain the coordinates (*x*, *y*) of each point. These values were introduced into a software developed in our laboratory to assess the locomotion speed and kinematics analyses. Each of the steps of the mice captured on the video was analyzed separately.

### Step Classification

We selected and analyzed five to seven steps in the left and right hindlimbs in each mouse. Because of the diversity of steps observed between and within groups, we evaluated the distribution of types of steps for each group before and after the injury. We classified them into Type A: the step was equal in time in both hindlimbs. Type B steps were those in which the mice took shorter steps with both hindlimbs, whereas Type C were the steps in which left hindlimb stride was longer than the right stride, and Type D steps were the opposite: right hindlimb stride was longer than the left one.

### Locomotion Speed Analysis

We determined the speed of the step (left and right) using software developed in our laboratory, which allows us to evaluate treatment effectiveness. A control group was defined using the measurements of all the steps of all mice before the injury. Each of the steps of the mice was analyzed separately. For each step, the length and speed of the step were calculated using the *x* coordinates (horizontal axis) of the metatarsus. We analyzed the step speed statistics of all the groups with and without considering the kind of steps and performed statistical hypothesis tests to evaluate the differences in the distributions of the groups.

### Displacement Analysis

To assess locomotion kinematics pattern analysis, we generated displacement curves on the horizontal and vertical axes with respect to time for each point of interest described before. All curves were normalized with respect to the step using a value range from 1 to 100 employing a spline-based interpolation.

**Figure 1:**
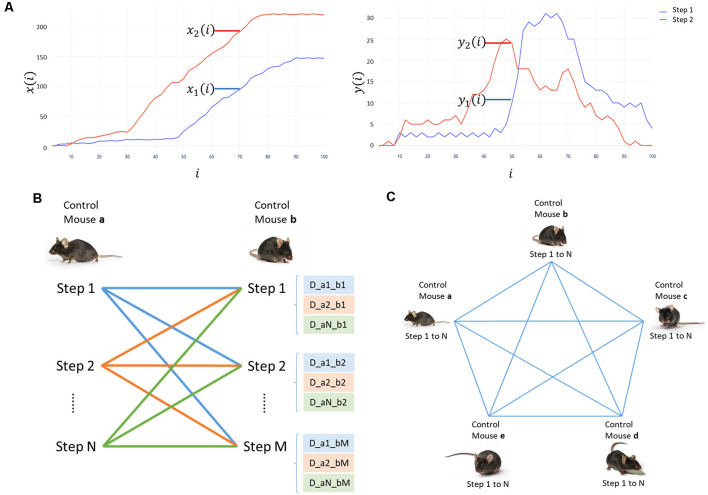
Analysis process for the displacement data. **(A)** Displacement graph in *x* and *y* for step 1 (red) and step 2 (blue). *x(i)* corresponds to horizontal displacement and *y(i)* to vertical displacement. The percentage of the step cycle (*i*) is in the abscissa. The coordinates in millimeters are graphed on the ordinate. **(B)** Scheme of the comparison of all steps in mouse “a” with all steps in mouse “b.” The distance between the displacement curves of in each step are writtem as “D_aN_bM.” **(C)** Scheme of the comparisons between all mice in the control group to determine the minimum and maximum distances of each mouse.

To determine the kinematic changes between groups, we compared the displacement curves among all studied mice. The difference between the curves was calculated using the Euclidean distance between each of the points of the normalized curve on the horizontal (*x*) and vertical (*y*) directions as follows:

$$D_{<1,2>}=\frac{1}{200}\sqrt{\sum_{i=1}^{100}{\left(x_{1}\left(i\right)-x_{2}\left(i\right)\right)}^{2}+\sum_{i=1}^{100}{\left(y_{1}\left(i\right)-y_{2}\left(i\right)\right)}^{2}}$$

where *D*_<1,2>_ is the squared error between every point of the normalized curves, defined as distance (*D*); “*x_1_ (i) − x_2_ (i)*” is the difference (*d*) between the coordinates in *x*, and “*y_1_ (i) − y_2_ (i)*” in *y* of every point in the graph, when comparing two steps (1 and 2; Figure 1A); and “*i*” is the percent in the step cycle.

We compared all the curves of every mouse in the control group. Every step curve of a mouse (a) was compared to the curves of a different control mouse (b; Figure 1B), and sequentially with each displacement curve of all mice in the control group (Figure 1C). For each mouse, we selected two pairs of curves: the most similar ones to calculate the minimum distance (*D*_Min_); and the most divergent ones to calculate the maximum distance (*D*_Max_). These values indicate the minimum and maximum distances between the steps in control. We used those as a reference of the expected results and to contrast them with the distances obtained when comparing mice in other groups with controls.

We compared every step curve of the mice in CI, HI, and HIEV with each displacement curve of mice in the control group and selected the two pairs of curves mentioned before per mouse. Thus, we calculated the minimum (*D*_Min_) and maximum (*D*_Max_) differences for every mouse when comparing with a control mouse. *D*_Min_ values comparison will demonstrate the differences observed when we contrast the most alike curves in all groups, whereas *D*_Max_ values analysis described the differences in the curves that are already different between each other. We graphed these values in a box plot and performed statistical hypothesis tests to evaluate the differences in the distributions of the groups.

### Histological Analysis

At 7 DPI, mice were anesthetized with xylazine/ketamine [intraperitoneal (IP), 80 mg/kg] and intracardially perfused with phosphate-buffered saline (PBS) and fixated with 4% paraformaldehyde in PBS. The brain was immediately removed and postfixed for 48 h at 4°C. The paraffin-embedded area of interest (−1.5 to −2.5 mm from Bregma) was sliced into 5-μm-thick sections. Approximately 40 slices were obtained and mounted into positively charged slides. We used hematoxylin–eosin staining for assessing the extension of the penetrating brain injury. A representative sample of nine brain sections was rehydrated in alcohol solutions and then submerged in a 5% hematoxylin solution for 3 min, followed by a 2% eosin solution for 30 s. Slides were finally washed, dehydrated, and sealed with Entellan resin. We observed the brain tissue slices using a light microscope. We examined the ipsilateral and contralateral hemispheres. We measured the depth of the penetrating injury and evaluated the lesion. We observed the neuronal morphological status in the brain tissues of the HI and HIEV groups. The area of the lesion was determined following the brain injury trajectory and considering an area of 0.04 mm^2^ from the center of the lesion. The percentage of pyknotic cells was calculated counting the cells located in the lesion area of CA1, CA2, and DG, in the hippocampus. We used ImageJ software to analyze brain tissue images.

### Statistical Analysis

Statistical analyses were carried out in the Minitab 19.2 and GraphPad Prism 8 software. We performed nonparametric Kruskal–Wallis and Wilcoxon tests in behavioral and neurological examinations. For the remaining analyses, multiple *t*-tests and Bonferroni *post hoc* analysis were employed. In all cases, the statistically significant value was taken when *p* < 0.05. We expressed all data as mean ± SD.

## Results

### eMSC–EV Characterization

**Figure 2:**
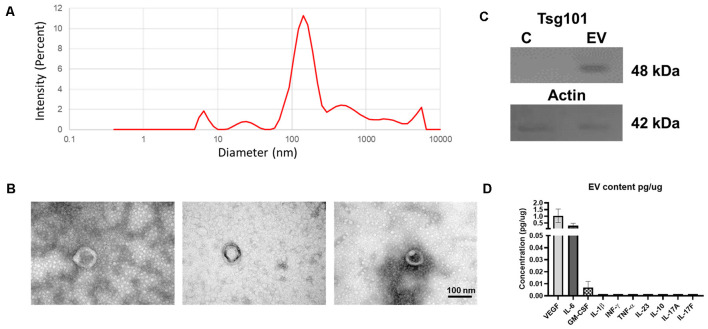
Characterization of endometrial mesenchymal stem cells-extracellular vesicles (eMSC–EVs). **(A)** Graph of dynamic light scattering (DLS) illustrating particle size of the EVs. The percentage of intensity is in the ordinate and the diameter of the particles in nanometers in the abscissa. The graph shows the mean of three independent experiments. **(B)** Transmission electron microscopy (TEM) images of eMSC–EVs show one EV in each panel. Scale bar: 100 nm. **(C)** Western blot of EVs marker Tsg101 (48 kDa) in the upper panel and actin (49 kDa) is in the lower panel. C, control; EV, extracellular vesicle sample. **(D)** Multianalyte profiling results of the eMSC–EV content. Biomarkers found in the eMSC–EV samples. We determine the presence of vascular endothelial growth factor (VEGF), IL-6, and granulocyte-macrophage colony-stimulating factor (GM-CSF). No detectable levels of IL-23, IL-10, IL-1β, TNF-α, IFN-γ, IL-17A, or IL-17F were found. The ordinate shows the concentration of each biomarker in pg/μg. The abscissa shows all the molecules analyzed. Graph represents the means and SD of three independent experiments.

The eMSCs obtained here were adherent to the culture plate and have the characteristic spindle shape. Those cells were positive for CD105, CD73, and CD90 cell markers and negative for hematopoietic CD45 and CD34 markers (data not shown). EVs secreted by these cells were isolated, with a multistep centrifugation and ultrafiltration method. eMSC–EV fractions were characterized according to current standards. DLS of three independent samples showed a peak diameter size at 100–200 nm (Figure 2A). In the sample, we observed round bodies of 100 nm by TEM (Figure 2B). We demonstrated the expression of Tsg101 in eMSC–EVs extracts using Western blot (Figure 2C). MAP results of the eMSC–EVs determined the presence of the growth factors VEGF and GM-CSF at 1.04 ± 0.49 and 0.0072 ± 0.004 pg/μg of protein, respectively. IL-6 was also found at 0.30 ± 0.18 pg/μg of protein (Figure 2D).

### Neurological Outcomes of the Penetrating Hippocampal Injury Model

**Figure 3:**
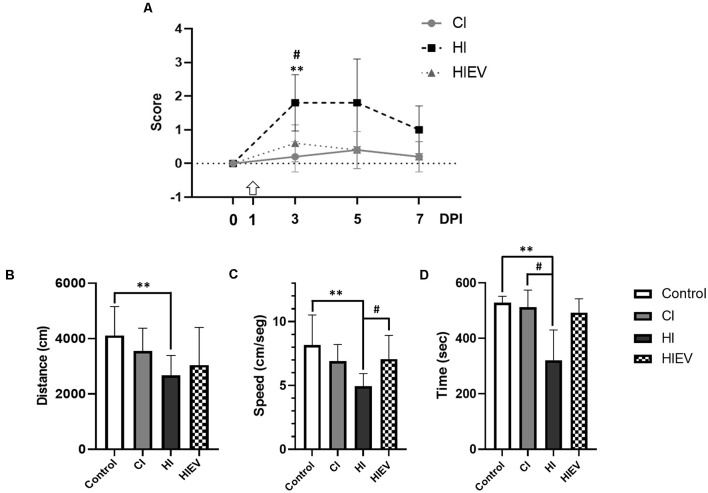
Mice neurological and behavioral outcomes in control, cortex-injured (CI), hippocampus-injured without treatment (HI), and hippocampus-injured with EV treatment (HIEV) groups. **(A)** Neurological Severity Score (ordinate) at 0, 3, 5, and 7 Days Post-Injury (DPI; abscissa). Symbols above the curves indicate statistically significant differences among HI vs. CI (**p < 0.01) and HI vs. HIEV (^#^*p* < 0.05). The arrow represents the day of eMSC–EV administration. OF test: distance traveled, running speed, and time spent in periphery (ordinates) in panels **(B–D)**, respectively, in the different groups (abscissas). In all graphs, the black lines above the columns indicate significant statistics between groups. Symbols above the black lines indicate differences with the control (**p < 0.01) or HI (^#^*p* < 0.05) group. Graphs represent the means and SD of five animals in each group. CI, cortex-injured group; HI, hippocampal-injured group; HIEV, hippocampal-injured group with eMSC–EV treatment.

In hippocampal injury, the neurological score at 3 DPI was 1.8 ± 0.8. In the cortex lesion, the score was 0.2 ± 0.4, and the statistical differences were significant with a *p* < 0.01. eMSC–EV administration significantly improved the neurological score to 0.4 ± 0.5, with statistical significance (*p* < 0.05). At 5 and 7 DPI, the score returned to control value in all groups (Figure 3A).

In the OF, we tested mice before the injury (Day 0) as a control group for further comparisons. Figure 3B illustrates that the control group traveled a total distance of 4,113.2 ± 1,044.1 cm; CI, 3,554.0 ± 823.4 cm; and HI, 2,671.6 ± 718.8 cm. We found statistically significant differences when comparing the HI and control groups (*p* < 0.01). eMSC–EV administration did not improve this parameter. The control group running speed was 8.2 ± 2.3 cm/s. In the cortex and hippocampus injury group, it was 6.9 ± 1.3 and 4.9 ± 0.9 cm/s, respectively. A statistically significant difference was observed between the control and HI groups (*p* < 0.01). eMSC–EV-administered group’s running speed was 7.1 ± 1.8 cm/s, statistically significant in comparison with the HI group (*p* < 0.05), see Figure 3C. We evaluated thigmotaxis and fecal boli production in all groups. Control mice spent an average of 528.3 ± 23.2 s in the periphery of the field, similar to what was observed for CI mice (512.9 ± 61.2 s). In HI mice, this behavior decreased to 320.3 ± 110.1 s. Statistically significant differences were detected when compared to the HI vs. control (*p* < 0.01) and HI vs. CI (*p* < 0.05) groups (Figure 3D). The average time in HIEV mice was 492.8 ± 50.2 s, with no statistical differences when compared with the HI group. There were no statistical differences between any groups in fecal boli production.

### Locomotion Speed Analysis

**Figure 4:**
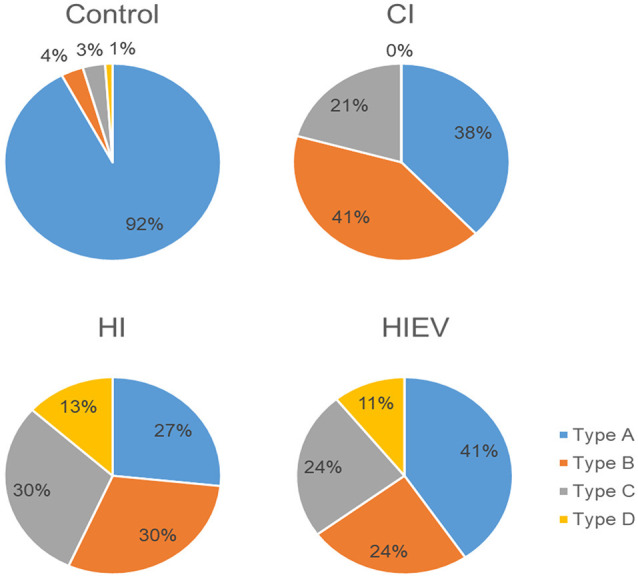
Mice step type distribution. We analyzed the left and right hindlimbs in every step. We analyze five to seven steps per mouse. Type A: the steps were equal in time in both hindlimbs. Type B: the steps were shorter with both hindlimbs. Type C: left hindlimb stride was longer than the right one. Type D: right hindlimb stride was longer than the left one. CI, cortex-injured group; HI, hippocampal-injured group; HIEV, hippocampal-injured group with eMSC–EV treatment.

We classified mice steps, as stated in *Step Classification*. In control animals, the type of steps with the highest percentage was A. In the HIEV group, Type A steps were predominant. In the CI, the dominant type was B, and in HI were B and C (Figure 4). As Type A was predominant in the control group, we decided to analyze those steps in all groups.

**Figure 5:**
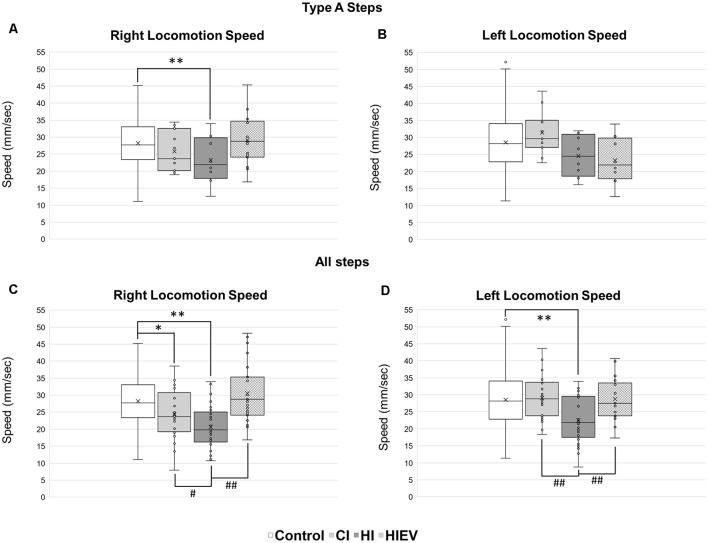
Locomotion speed analyses of steps in control, HI, HIEV, and CI groups. Locomotion speed of Type A steps in the **(A)** right and **(B)** left hindlimbs. Locomotion speed of all steps in the **(C)** right and **(D)** left hindlimbs. In the ordinates, the axis is the locomotion speed values of every step analyzed of each mouse in mm/s. In abscissa, different groups. Symbols above the black lines indicate differences when comparing any group with the control (*p < 0.05, **p < 0.01) or HI (^#^*p* < 0.05, ^##^*p* < 0.01) group. CI, cortex-injured group; HI, hippocampal-injured group; HIEV, hippocampal-injured group with eMSC–EV treatment.

In Type A steps, we found a significant reduction of speed in the right hindlimb of the HI group (23.23 ± 7.13 mm/s) in comparison with the control group (28.20 ± 7.09 mm/s; *p* < 0.01; Figure 5A). Steps speed differences did not occur in the left hindlimb (Figure 5B). As differences between all steps were step cycle duration, we decided to analyze the speed considering all types of steps of each mouse.

Considering all steps, in the right hindlimb, control, CI, and HI locomotion speed were 27.72 ± 7.15, 24.38 ± 7.04, and 20.66 ± 6.4 mm/s, respectively (Figure 5C). We observed statistically significant differences when comparing the control group with the HI (*p* < 0.01) and CI (*p* < 0.05) groups. The speed in the right hindlimb of the HIEV group was 30.53 ± 8.79 mm/s. We found significant differences when compared to the HI vs. HIEV (*p* < 0.01) and HI vs. CI (*p* < 0.05).

In the left hindlimb, the control group speed was 27.87 ± 8.14; in the CI group, 29.09 ± 6.05; and the HI group speed was 21.94 ± 6.61 mm/s. The differences among the HI vs. control and HI vs. CI groups were statistically significant (*p* < 0.01) in this hindlimb. The decrease in the speed was reversed after the administration of eMSC–EVs: in the HIEV group, the speed was 28.75 ± 6.02 mm/s. No statistical differences occurred when the treated group was compared with control. A significant difference was found between the HI and HIEV (*p* < 0.01) groups (Figure 5D).

The locomotion speed reduction seen in the HI group could not be attributed to a decrease in the step length, because the step lengths considering all types of steps in all groups did not have differences (data not shown).

### Displacement Analysis

**Figure 6:**
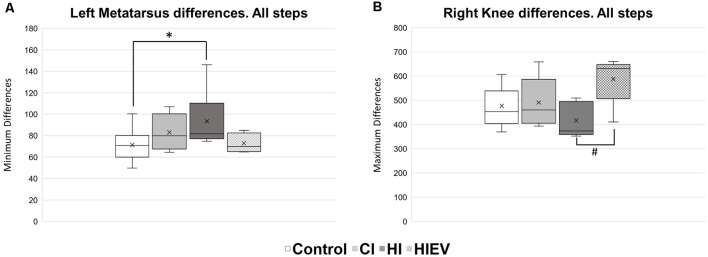
*D*_Min_ and *D*_Max_ values analysis of all steps in control, HI, HIEV, and CI groups. **(A)** *D*_Min_ values of left metatarsus curves in all steps. **(B)** *D*_Max_ values of right knee curves in all steps. *D*_Min_or *D*_Max_ are graphed in the ordinates. The abscissas show the different groups. Symbols above the black lines indicate differences when comparing any group with the control (*p < 0.05) or HI (^#^*p* < 0.05) group. CI, cortex-injured group; HI, hippocampal-injured group; HIEV, hippocampal-injured group with eMSC–EV treatment.

We evaluated *D*_Min_ and *D*_Max_ of each mouse considering all and Type A steps at 7 DPI. We analyzed *D*_Min_ values first and considered all steps without classification. We found significance only when comparing the left metatarsus curves of the control vs. HI groups (*p* < 0.05; Figure 6A). We did not observe *D*_Min_ differences between the curves of the groups in the other points of interest.

In respect to *D*_Max_ values, we found significance when comparing all steps in the curves of the right knee between the HI and HIEV groups (*p* < 0.05), as seen in Figure 6B.

**Figure 7:**
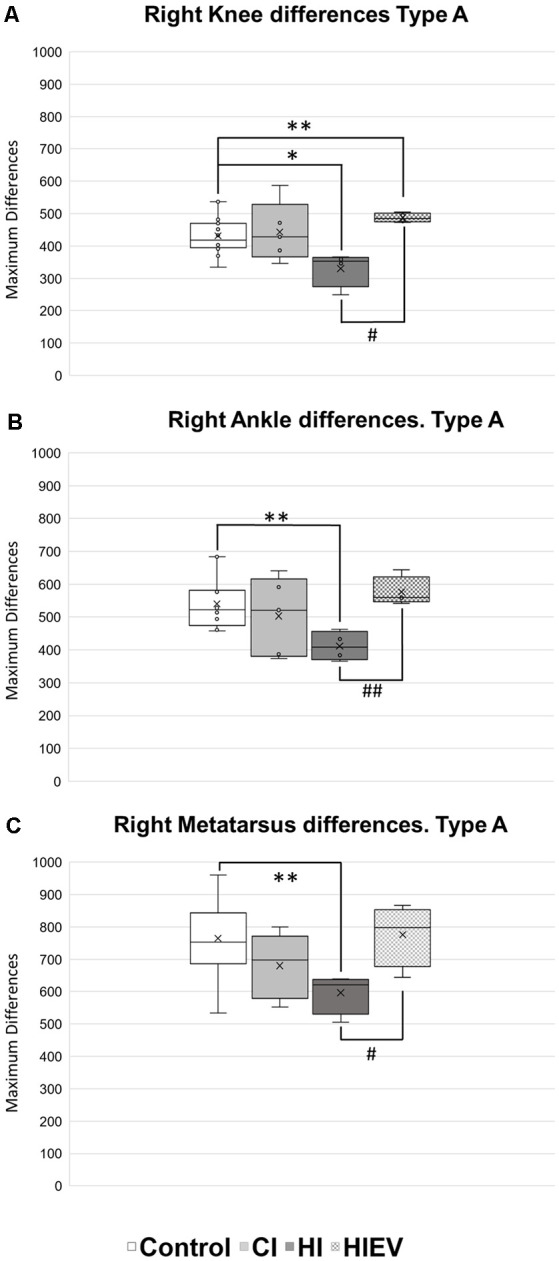
*D*_Max_ values of Type A steps of right hindlimb points of interest. **(A)** Knee differences, **(B)** ankle differences, **(C)** metatarsus differences. *D*_Max_ are graphed in the ordinates. The abscissas show the different groups. Symbols above the black lines indicate differences when comparing any group with Control (*p < 0.05; **p < 0.01) or HI (^#^*p* < 0.05, ^##^*p* < 0.01) group. CI, cortex-injured group; HI, hippocampal-injured group; HIEV, hippocampal-injured group with eMSC–EV treatment.

We decided to analyze displacement curve differences between Type A steps too, but we did not observe differences when comparing *D*_Min_ values. We observed improvement of HIEV groups and differences between the control and HI groups in all points of interest. The right knee comparisons showed statistically significant differences when comparing the control with HI (*p* < 0.05) and HIEV (*p* < 0.01) groups. Significant improvements were observed when EVs were administered and in comparison with the hippocampal injury group (*p* < 0.05), as seen in Figure 7A. In the right ankle (Figure 7B), we observed differences between the control and HI groups (*p* < 0.01). Also, we found lower *D*_Max_ values in HI group compared with the HIEV (*p* < 0.01). In Figure 7C, lower *D*_Max_ values were observed in right metatarsus curves of HI groups compared with the control (*p* < 0.01) and HIEV groups (*p* < 0.05).

**Figure 8:**
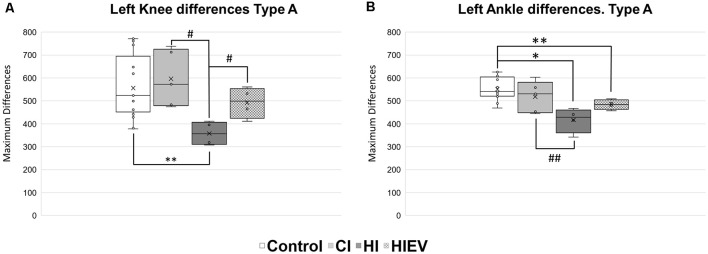
*D*_Max_ values of Type A steps of left hindlimb points of interest. **(A)** Knee differences, **(B)** ankle differences. *D*_Max_ are graphed in the ordinates. The abscissas show the different groups. Symbols above the black lines indicate differences when comparing any group with Control (*p < 0.05; **p < 0.01) or HI (^#^*p* < 0.05, ^##^*p* < 0.01) group. CI, cortex-injured group; HI, hippocampal-injured group; HIEV, hippocampal-injured group with eMSC–EV treatment.

HI *D*_Max_ values in left knee were lower when compared with the control (*p* < 0.01), CI (*p* < 0.05), and HIEV (*p* < 0.05) groups (Figure 8A). In the left ankle (Figure 8B), *D*_Max_ values showed differences when comparing the control with HI (*p* < 0.05) and HIEV groups (*p* < 0.01). The HI and CI groups also presented statistically significant differences (*p* < 0.01). The left metatarsus *D*_Max_ graph showed no statistically significant differences between groups (data not shown).

### Histological Analysis of the Hippocampus

**Figure 9:**
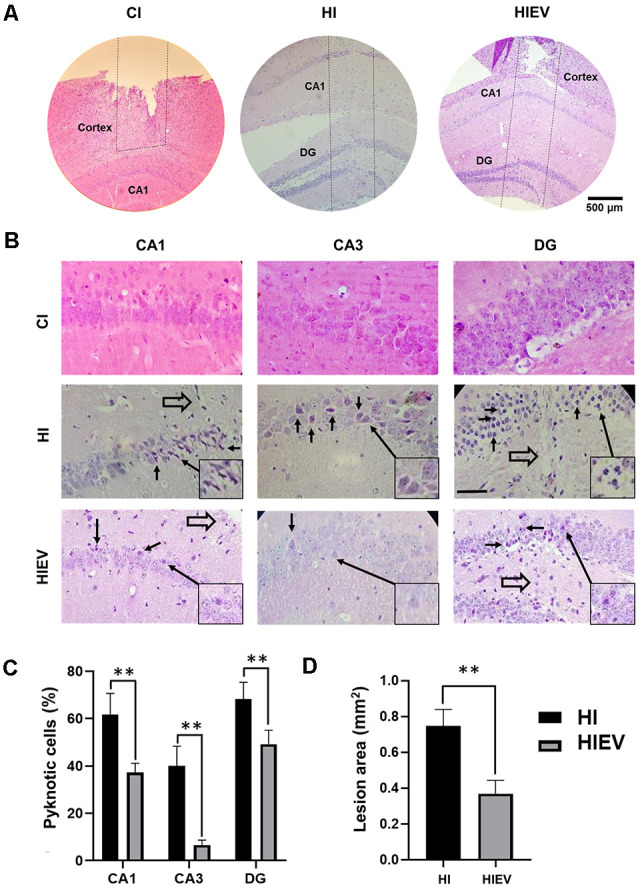
Histological analysis of ipsilateral cells and the damaged area thought the hippocampus lesion at seven DPI. In CI, HI, and HIEV groups. **(A)** Penetrating injury trajectory is illustrated between the dotted lines. Note the depth in CI, HI, and HIEV groups at seven DPI. Scale bar = 500 μm. **(B)** Magnified CA1, CA3, and DG areas in CI, HI, and HIEV groups. Closed arrows indicate apoptotic cells and open arrows (CA1, DG), cells close to the trajectory of the lesion. Scale bar = 100 μm. In the frame, some pyknotic cells are magnified and indicated with a closed arrow. **(C)** Percentage of pyknotic cells (ordinate) in CA1, CA3, and DG areas (abscissa) of HI and HIEV groups. Note, the HI group had a higher percentage of pyknotic cells in all the analyzed areas. **(D)** The area in mm^2^ (ordinate) in HI and HIEV experimental groups (abscissa). **(C,D)** Graphs represent the means and SD of five animals of each group. The black lines above the columns indicate significant statistics (**p < 0.01) between the groups. CI, cortex-injured group; HI, hippocampal-injured group; HIEV, hippocampal-injured group with eMSC–EV treatment.

Cell and tissue loss occurred in the cortex of all groups (Figure 9). In the CI group, the hippocampus was not damaged, as shown in Figure 9A. In the HI and HIEV groups, the injury reached CA1 and DG. In both hippocampal-damaged groups, the dorsal but not ventral hippocampus was injured. For the histological analysis of the hippocampus, we considered only the HI and HIEV groups. Unidentified cells were found in the injury trajectory in the HI and HIV groups (Figure 9A). The percentage of pyknotic cells and injury areas was analyzed and compared. We observed neuronal cell damage in the ipsilateral hemisphere of the HI group. A high percentage of pyknotic cells were found in CA1, CA3, and DG (61.8% ± 8.8%, 40% ± 8.3%, and 68.2% ± 7.1%, respectively). In HIEV, this damage was more significant in CA1 (37.2% ± 3.9% of pyknotic cells) and DG (49.2% ± 5.9%), compared to CA3 (6.6% ± 2.1%), see Figure 9B. We observed more pyknotic cells in the HI group compared with the HIEV group (*p* < 0.01, Figure 9C). In the HI group, the lesion area in the hippocampus was 0.75 ± 0.09 mm^2^. In the HIEV group, it was 0.37 ± 0.07 mm^2^, given a statistically significant difference (*p* < 0.01) between the lesion areas of both groups (Figure 9D). Decreases in injury size and neuronal damage were observed after eMSC–EV administration. The contralateral hippocampal hemisphere was undamaged in the three groups (data not shown).

## Discussion

To the best of our knowledge, this is the first study to address quantitatively the locomotion speed and displacement in a mouse model of penetrating hippocampal injury, as well as the effect of eMSC–EVs in hippocampal damage through IN administration. We used only male mice because of the preponderance of the bibliography in male animal models. Besides, the estrus cycle in female mice could affect behavior (Xu et al., 2016; Wang et al., 2019), and it is difficult to evaluate the menstrual cycle phases or fluctuating sex hormones for precise comparisons. Stride length, mechanical behaviors, and changes in muscle strength during aging have been described between male and female mice due to sexual hormones (Moran et al., 2005; Bojados et al., 2013; Illien-Jünger et al., 2018). For this, in future studies, it is important to address female and male mice differences in kinematic analyses.

Neuronal apoptosis has been described after TBI in both humans and mice 24–48 h after the injury (Fox et al., 1998; Dreßler et al., 2007). In a mouse model of needlestick lesion, a very similar model of ours, gliosis and apoptosis are described 1 day after the damage (Purushothuman et al., 2013). Here, we determined the effect of EVs at 1 DPI. As our model also affects the cortex, we employed a cortical-injured group to analyze the effect of cortex damage alone.

NSS is one of the most used in animal studies. It proves sensorimotor impairments, with motor, equilibrium, and reflex tasks in animals with brain lesions (Schaar et al., 2010; Zarruk et al., 2011). We did not observe sensorimotor impairments in the cortex-injured mice, compared with the control. However, it was noticeable that the HI group had higher scores, compared with all groups. Mice treated with eMSC–EVs after the hippocampal penetrating injury demonstrated reduced sensorimotor deterioration and faster neurological recovery. Despite that we observed recovery in the HI group, this was not completed at 7 DPI. Therefore, spontaneous recovery in eMSC–EV-treated mice can be ruled out.

OF test has been extensively used to measure behavior and locomotion, as well as its relationship with hippocampus (Laghmouch et al., 1997). We measured the emotional state in our mice, with thigmotaxis and fecal boli production, as described before, to determine anxiety or fear (Choleris, 2001). We did not find differences in fecal boli production in any groups analyzed. We found a time reduction in the periphery in mice with hippocampus injury compared with the control group. This could be associated with a diminished anxiety behavior, as it is observed with an anxiolytic drug (Choleris, 2001). There were significant differences between the CI and HI groups in thigmotaxis. This suggests that the behavioral change is mainly due to the hippocampal injury. Similar to what we found, TBI mice with cortex damage did not present any behavioral changes after the injury (Niesman et al., 2014). Moreover, we observed damage only in the dorsal region of the hippocampus. It has been suggested that this region has a main role in spatial memory; meanwhile, ventral region is more involved in sensorial responses and anxiety (Ferbinteanu and McDonald, 2000; Fanselow and Dong, 2010; Bannerman et al., 2014). Despite that any specific hippocampal region has been linked to locomotion, there are several studies in which dorsal hippocampus is modified that associate hippocampus with speed locomotion (Sławińska and Kasicki, 1998; Bender et al., 2015; Hernández-López et al., 2017).

OF test has been used to analyze motor behaviors, mainly locomotor activity. It has been used to study disease models that involve muscular strength or movement capacity impairments (Seibenhener and Wooten, 2015). We found that locomotor activity in all groups was preserved, despite the injuries. However, locomotor speed was reduced in the HI group compared with the control and HIEV groups. The results in behavior and locomotor speed decreased after EV administration suggests that functional recovery could be expected with eMSC–EV IN administration. Behavioral tests, such as OF, provide information about general motor performance and anxiety that could not be separated from each other (Mendes et al., 2015; Rostosky and Milosevic, 2018). To establish the hippocampal role in locomotor control, kinematic analyses are needed (Rostosky and Milosevic, 2018).

We tested the locomotion kinematics of mice using a tunnel walk test. We studied the left and right hindlimbs of every step. In the cortex-injured group, we observed a reduction of step speed only in the right hindlimb when comparing all steps without classification, whereas the injury was made in the left hemisphere. In agreement, the corticospinal tract that originated in the left side in mammals participated in the control of movements in the right side due to the pyramidal decussation at the brainstem level (Vulliemoz et al., 2005; Welniarz et al., 2017). The cortex injury in this work affected primarily sensorimotor areas. However, there is a complex topology of brain networks in the mouse brain (Rubinov et al., 2015). Sensory and motor functions overlap to produce adequate hindlimb movements (Hall and Lindholm, 1974). Also, the connections between S1 and M1 areas are already known (Chakrabarti and Alloway, 2006). For this, motor impairments could be expected in the CI group.

After the CA1 layer was damaged in the HI group, speed reduction was evident in both hindlimbs. In mice, CA1 sensibilization to afferent inputs regulates the running speed (Fuhrmann et al., 2015). Therefore, we can probably presume that, in our studied mice, the speed modulation capacity of the hippocampus was lost. Changes in locomotion speed in a rat model of penetrating hippocampal injury due to CA1 damage were previously described (López Ruiz et al., 2015). Slow walking speed was correlated with functions associated with the hippocampus: impaired processing speed, executive function, and episodic memory (Kim and Won, 2019). Patients with hippocampus pathologies such as Alzheimer disease also experience episodic memory and executive function impairments (Burgess et al., 2002). Hippocampal volume loss is correlated with slower processing speed (Papp et al., 2014; O’Shea et al., 2016) and poorer performance in episodic memory and executive functions (O’Shea et al., 2016). The locomotion speed reduction after hippocampal injury was reversed with eMSC–EV administration. Differences between cortex and hippocampus damage in left hindlimb speed locomotion reveal the different effects of the injured structures.

Increased variability in the type of steps in the cortex-injured group was observed, compared with the control group. Step variability seems to increase when the hippocampus compensates the damage in other brain regions (Beauchet et al., 2019). Our mice presented walk pattern variability, as we observed four different steps. Matching our results, stride abnormalities in individuals with dementia or cognitive impairments included decrease in walking speed (IJmker and Lamoth, 2012; Doi et al., 2014) and walk variability (Montero-Odasso et al., 2009; IJmker and Lamoth, 2012; Doi et al., 2014; Beauchet et al., 2019; Zhang et al., 2019). Our findings could be related to clinic symptoms, as hippocampal damage produces walk speed and variability changes in both humans and mice. In elderly people, dual test showed that walking may require more cognitive control than previously thought (Montero-Odasso et al., 2009; Lamoth et al., 2011). In these studies, it was demonstrated that cognitive functions are clearly associated with hippocampal integrity and with speed and step variability (IJmker and Lamoth, 2012; Doi et al., 2014). There are many characteristics shared between neurodegenerative diseases and the chronic lesion of TBI, including inflammatory and neurovascular pathologies (Purushothuman et al., 2013; Zhou et al., 2020). The lesion described here and the effects in kinematics could be useful for understanding several human diseases.

We did not observe changes in the displacement curves of the CI group with respect to the control group. We report differences between the displacement curves of HI group only in mice metatarsus when comparing *D*_Min_ in all steps. In accordance to our findings, metatarsal curve differences were reported in a mouse with hippocampal accumulation of *Toxoplasma* cysts (Galván-Ramírez et al., 2019). Knee *D*_Max_ differences were evident between the mice with injury and the ones receiving eMSC–EVs. We observed that our method is sensible to step variation, because it did not identify differences with no step classification. When only comparing Type A steps, knee, ankle, and metatarsus displacement curve differences were evident after hippocampal damage in comparison with control group, but only when we compared the most dissimilar curves (*D*_Max_). This analysis reveals the variability between group’s displacement curves. In all points of interest, the hippocampal-injured mice displacement variability decreases. When hippocampus is damaged, locomotion speed decreases due to alterations of gamma oscillations (Chen et al., 2011). In the HI group, this wave could be altered, and the variation reduction observed could be because these waves are associated with decision making, attention, processing speed, and executive functions and thus the loss of spatial navigation (Ahmed and Mehta, 2012).

We found displacement differences in both hindlimbs in the hippocampal injury group. In rats, the reported changes were only in contralateral hindlimb (López Ruiz et al., 2015). Despite penetrating hippocampal injury was similar in both of our models, there are anatomical and physiological divergences between both rats and mice (Ellenbroek and Youn, 2016). The injury in each species model could affect different pathways that control locomotion in each species and present different results. To address this, a comparative study must be carefully performed.

It has been shown that MSC–EVs can ameliorate impaired hippocampal functions (Deng et al., 2017) and restore synaptic transmission in Schaffer collateral to CA1 synapses (Wang et al., 2018). Hence, the locomotion speed preservation in the eMSC–EV-administered group could be due to CA1 preservation, which in turn could be related to functional recovery, as seen before (López Ruiz et al., 2015). Displacement curves in EV-treated mice returned variability to control values, and in some cases, it became greater. In general, administration of eMSC–EVs improved the outcome of all tests evaluated. In the histology analysis, we observed lower percentage of pyknotic cells and less damaged area in the hippocampus at 7 DPI after the application of eMSC–EVs in comparison with the nontreated group. This is similar to that reported before in TBI (Li et al., 2017; Ni et al., 2019), stroke (Chen et al., 2016), or Alzheimer disease (Cui et al., 2019; Elia et al., 2019) models.

We identified three factors in eMSC–EVs: VEGF, GM-CSF, and IL-6, in accordance to what is reported for MSC–EVs from different origins (Meng et al., 2007; Alcayaga-Miranda et al., 2015; Lai et al., 2015; Lee et al., 2016). These molecules are related to angiogenesis and neurogenesis and could promote the expression of other neurotrophic factors with neuroprotective effects in the hippocampus (Zhang et al., 2013; Perígolo-vicente et al., 2014; Khan et al., 2015; Sun et al., 2017; Yan et al., 2017; Dougan et al., 2019). Damage prevention of MSC–EVs was observed previously in a TBI rat model, in which the early application reduced the brain inflammatory state and induced cognitive rescue (Kim et al., 2016; Ni et al., 2019). Further studies are needed to determine whether eMSC–EV treatment promotes angiogenesis, neurogenesis, or vascular remodeling, as well as the pathways involved in these mechanisms. In comparison with our study, IN administration of EVs from bone marrow-derived MSCs demonstrated positive effects in the hippocampus (Losurdo et al., 2020).

One limitation of this study is that we did not test the effect of different dosage of eMSC–EVs on hippocampus damage prevention. Future work is required to elucidate the mechanisms of the protective and restorative effects of exosomes and to optimize the dosage and treatment regimen.

## Conclusion

Penetrating hippocampal injury generates neuron loss in the CA1 region, probably disrupting a network involved in locomotion speed modulation that caused locomotion speed reduction. Displacement changes were also present in mice with hippocampal injury, probably due to loss of spatial navigation. The IN administration of eMSC–EVs after hippocampal injury prevented histological damage and preserved speed locomotion and displacement changes presumably due to the growth factors contained in those vesicles. Our results provide evidence to consolidate the view that the hippocampus plays an essential role in locomotion speed and navigation in rodents and suggest that eMSC–EV IN administration could improve recovery of hippocampal tissue.

## Data Availability Statement

The raw data supporting the conclusions of this article will be made available by the authors, without undue reservation.

## Ethics Statement

The animal study was reviewed and approved by “Comité Institucional de Cuidado y Uso de Animales de Laboratorio (CICUAL),” affiliated to the University of Guadalajara and endorsed to “Comisión Nacional de Bioética” with the number CONBIOÉTICA-14-CEI-002-20191003.

## Author Contributions

LL-M, RC-A, JR-C, and SD-J contributed to the conception and design of the study. RC-A, JR-C, and SD-J supervised all experiments and data obtention. GM-R and CA-P developed and provided statistical validation to the quantitative kinematics analysis. LL-M, IA-G, and LN-A standardized relevant methods for the experiments of the project. LL-M, ET-A, MD-D, and CG-A contributed to the data obtention, curation, and the formal analysis of the data. LL-M wrote the first draft of the manuscript. RC-A, JD-J, and SD-J revised and edited the manuscript. JR-C obtained financial support for the project. All authors contributed to the article and approved the submitted version.

## Conflict of Interest

The authors declare that the research was conducted in the absence of any commercial or financial relationships that could be construed as a potential conflict of interest.

## Abbreviations

DLS: Dynamic Light Scattering
DPI: Days Post-Injury
eMSC: Endometrial Mesenchymal Stem Cells
eMSC–EVs: Endometrial Mesenchymal Stem Cells derived Extracellular Vesicles
EVs: Extracellular Vesicles
IN: Intranasal
MSCs: Mesenchymal Stem Cells
mNSS: Modified Neurological Severity Score
TBI: Traumatic Brain Injury
TEM: Transmission Electron Microscopy.
